# IPD072Aa from Pseudomonas chlororaphis Targets Midgut Epithelial Cells in Killing Western Corn Rootworm (*Diabrotica virgifera virgifera*)

**DOI:** 10.1128/aem.01622-22

**Published:** 2023-02-27

**Authors:** Nuria Jiménez-Juárez, Jarred Oral, Mark E. Nelson, Albert L. Lu

**Affiliations:** a Corteva Agriscience, Johnston, Iowa, USA; Norwegian University of Life Sciences

**Keywords:** *Pseudomonas chlororaphis*, *Diabrotica virgifera virgifera*, rootworm, insect, bacterial toxin, confocal microscopy, electron microscopy, biotechnology

## Abstract

IPD072Aa from Pseudomonas chlororaphis is a new insecticidal protein that has been shown to have high activity against western corn rootworm (WCR). IPD072 has no sequence signatures or predicted structural motifs with any known protein revealing little insight into its mode of action using bioinformatic tools. As many bacterially derived insecticidal proteins are known to act through mechanisms that lead to death of midgut cells, we evaluated whether IPD072Aa also acts by targeting the cells of WCR midgut. IPD072Aa exhibits specific binding to brush border membrane vesicles (BBMVs) prepared from WCR guts. The binding was found to occur at binding sites that are different than those recognized by Cry3A or Cry34Ab1/Cry35Ab1, proteins expressed by current maize traits that target WCR. Using fluorescence confocal microscopy, immuno-detection of IPD072Aa in longitudinal sections from whole WCR larvae that were fed IPD072Aa revealed the association of the protein with the cells that line the gut. High-resolution scanning electron microscopy of similar whole larval sections revealed the disruption of the gut lining resulting from cell death caused by IPD072Aa exposure. These data show that the insecticidal activity of IPD072Aa results from specific targeting and killing of rootworm midgut cells.

**IMPORTANCE** Transgenic traits targeting WCR based on insecticidal proteins from Bacillus thuringiensis have proven effective in protecting maize yield in North America. High adoption has led to WCR populations that are resistant to the trait proteins. Four proteins have been developed into commercial traits, but they represent only two modes of action due to cross-resistance among three. New proteins suited for trait development are needed. IPD072Aa, identified from the bacterium Pseudomonas chlororaphis, was shown to be effective in protecting transgenic maize against WCR. To be useful, IPD072Aa must work through binding to different receptors than those utilized by current traits to reduce risk of cross-resistance and understanding its mechanism of toxicity could aid in countering resistance development. Our results show that IPD072Aa binds to receptors in WCR gut that are different than those utilized by current commercial traits and its targeted killing of midgut cells results in larval death.

## INTRODUCTION

Corn rootworms (CRW) are a major pest of maize in North America and an increasing problem in Europe since their introduction (1). WCR is currently the predominant species in the central maize growing areas of the United States, estimated to cost growers billions of dollars annually in yield loss and control measures (1). Maize hybrids expressing transgenes that target WCR have been an effective pest management tool available to growers in North America allowing a reduction in chemical insecticide use. All CRW traits introduced to date have been based on genes from Bacillus thuringiensis. These traits have consisted of several modified Cry3 proteins (Cry3Bb1, mCry3Aa, and eCry3.1Ab) as well as the binary insecticidal protein, Cry34Ab1/Cry35Ab1 (recently renamed Gpp34Ab1/Tpp35Ab1 under a revised nomenclature system (2)). The first CRW traits were deployed as single mode of action products, which required planting a significant percentage of a field to refuge plants (lacking an insect control trait) to decrease the selection pressure for resistant insects. Nevertheless, signs of decreased effectiveness led to the development of trait “pyramids” (individual plants expressing both a Cry3 protein along with the Gpp34Ab1/Tpp35Ab1 proteins) to provide two modes of action to help slow the development of resistance to the individual traits. In addition to the introduction of pyramids, blended or integrated refuge was shown to be better-suited than structured refuge for delaying resistance in WCR as it ensures refuge is planted in every Bt field and promotes mating between insects surviving on Bt and non-Bt plants (3). These improved resistance management tactics along with emphasis on integrated pest management has helped to prolong the effectiveness of existing CRW traits, but the continued reliance on Cry3 and Gpp34Ab1/Tpp35Ab1 proteins allows resistance to continue to evolve. Because of this, insecticidal proteins that represent different MoAs than the existing commercial traits are needed. The identification of new proteins from Bt has proven to be challenging, but recent reports of CRW-active proteins from non-Bt sources has been encouraging (4–7). The IPD072Aa protein identified from P. chlororaphis was shown to be effective against WCR that are resistant to Cry3 as well as Gpp34Ab1/Tpp35Ab1 proteins, indicating that it likely represents a new MoA (7). IPD072Aa also showed great efficacy in protecting maize plants from damage caused by CRW under greenhouse and field conditions. Here, we report data showing that the IPD072Aa protein kills WCR by disrupting the integrity of the epithelial lining of the larval gut. We show that IPD072Aa binds to sites in WCR midgut tissue that are different from those utilized by Cry3A and Gpp34Ab1/Tpp35Ab1 proteins providing direct evidence that it represents a new mode of action. Following ingestion, IPD072Aa associates with the enterocytes of the epithelium remaining localized within the larval gut. High-resolution microscopy reveals the histopathological impact of IPD072Aa on these cells that precedes larval death. Its toxicity can be attributed to the specific and selective targeting of the enterocytes that line the WCR gut as has been reported for Cry3 and Gpp34Ab1/Tpp35Ab1 proteins (8).

## RESULTS

### Sensitivity of IPD072Aa to WCR proteases.

**FIG 1:**
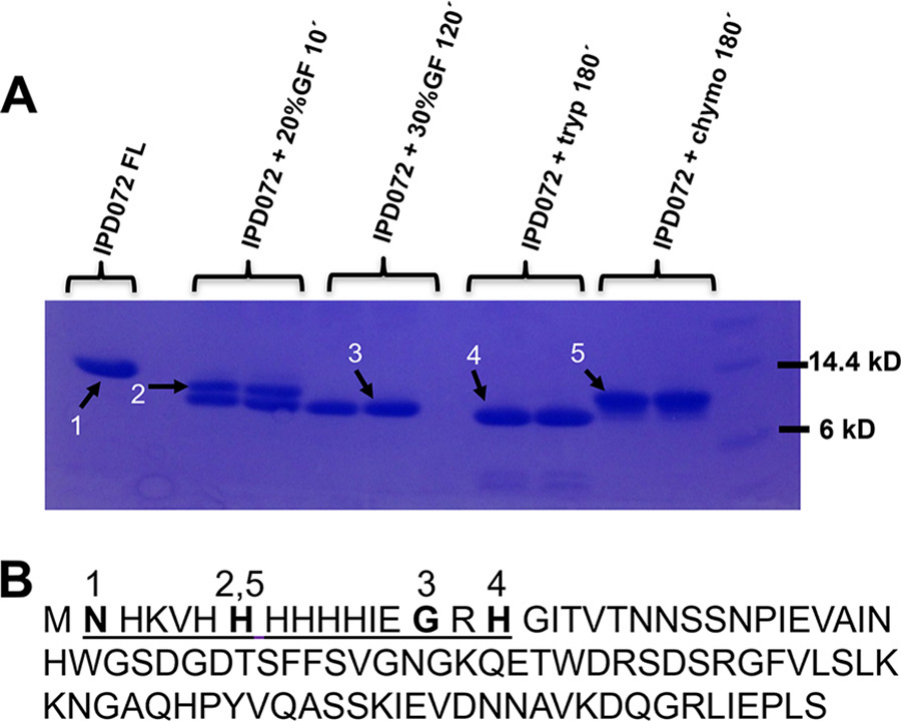
Digestion of IPD072Aa in the presence of midgut proteases. (A) IPD072Aa was incubated in the absence or presence of WCR GF to determine the size and N-terminus of the protein in its processed state. IPD072Aa was also treated with trypsin or chymotrypsin to determine if either could be used to mimic GF treatment. Aliquots of the protein were then separated by SDS-PAGE and transferred to PVDF membranes that were stained with Coomassie brilliant blue to identify digestion products to subject to Edman degradation sequence analysis. The labeling of the lanes reflects the following: IPD072FL is the untreated recombinant purified protein alone; +20%GF 10’ is the protein exposed to 20%GF for 10 min; +30%GF 120’ is the protein exposed to GF for 120 min; +tryp 180’ is the protein exposed to trypsin (1:50, w:w) for 180 min; +chymo 180’ is the protein exposed to chymotrypsin (1:50, w:w) for 180 min. IPD072FL was run in a single lane, while all treatments were run in duplicate. The white numbered black arrows identify the band sizes that were submitted for sequencing. (B) Edman degradation sequence analysis was used to determine the N-terminal amino acid of each fragment which is labeled above the full amino acid sequence of the protein. The N-terminal expression tag sequence is underscored. IPD072FL band 1 was missing only the initial M residue; +tryp 180’ band 4 completely removed the expression tag; +30% GF 120’ band 3 removed most of the expression tag, but two linker amino acids remained; +20% GF 10’ band 2 and +chym 180’ band 5 both cleaved within the 6×-His sequence near the middle of the expression tag.

Many insecticidal proteins undergo some degree of proteolysis when ingested by insect larvae. In some cases, this proteolysis is a necessary step in the mode of action of the protein as is the case for three-domain Cry toxins from Bt (9). To determine if such processing occurs for IPD072Aa, the recombinant purified protein with its N-terminal purification tag intact (16 amino acids, including the 6×-histidine) was incubated with gastric fluid (GF) collected from third instar WCR. The processing by GF was compared to the processing that occurs when IPD072Aa was exposed to commercially available trypsin and chymotrypsin to enable simplified processing *in vitro*, if needed. Mixtures of IPD072Aa and GF, trypsin, or chymotrypsin were prepared and incubated at room temperature. Aliquots of IPD072Aa were taken from the GF mixture at 10- and 120-minute time points, while trypsin and chymotrypsin were sampled at 3 h as longer durations resulted in the accumulation of no additional fragments. Theses aliquots were then subjected to SDS-PAGE to assess protein size shifts and then transferred to PVDF for Edman degradation sequencing (Fig. 1) to determine if and where the N-terminus was processed. Exposure to GF resulted in different degrees of removal of the expression tag, leaving three amino acids (GRH) from the purification tag after 2 h. Longer exposure to GF did not result in additional accumulation of any stable protein bands indicating that processing within the native IPD072Aa sequence is likely not required as part of its mode of action (data not shown). Exposure to trypsin for 3 h removed all but one amino acid of the purification tag, while exposure to chymotrypsin for 3 h was not effective in removing the purification tag.

### Specific binding of IPD072Aa to WCR brush border membrane vesicles.

To determine if IPD072Aa could bind specifically to the cells that line the WCR midgut, brush border membrane vesicles (BBMVs) binding was assessed. To monitor binding, IPD072Aa was covalently labeled with fluorescent dye, Alexa-Fluor488, which will be referred to as Alexa-IPD072. Differing amounts of Alexa-IPD072 were incubated with differing amounts of BBMVs in the absence and presence of excess unlabeled IPD072Aa in order to determine the conditions for optimal binding signal above background.

**FIG 2:**
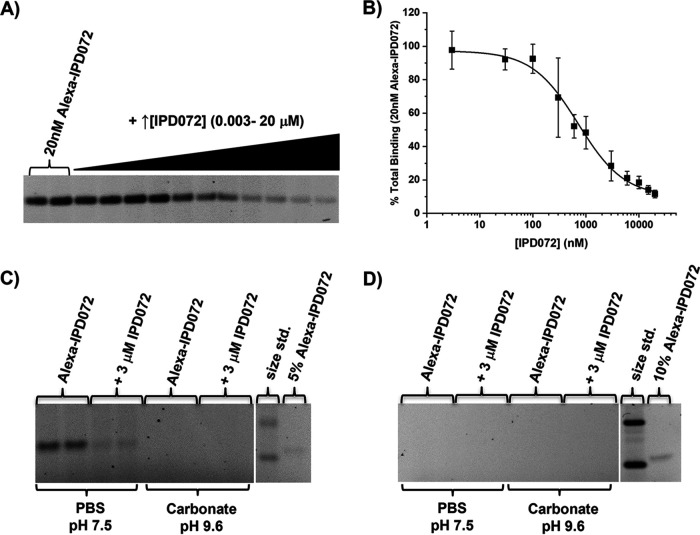
Specific binding of IPD072Aa to WCR BBMVs demonstrated by homologous competition, but no specific binding under conditions appropriate for binding to ECB BBMVs. (A) A representative gel image showing the fluorescent signal remaining from Alexa-IPD072Aa bound to WCR BBMVs as the concentration of unlabeled IPD072Aa was increased. (B) Shows the averaged densitometry values along with the standard deviations from three binding experiments normalized to the signal in the absence of unlabeled IPD072. The data were fit with a logistic equation to estimate the EC_50_ value which was 714 ± 120 nM. (C) shows a representative gel image from competitive binding experiments using Alexa-IPD072 (50 nM) and WCR BBMVs (20 μg) where specific binding was observed using a phosphate-buffered saline binding (PBS) buffer at near neutral pH, but not when using a carbonate buffer system at alkaline pH. (D) shows a representative gel image from competitive binding experiments using Alexa-IPD072 (50 nM) and ECB BBMVs (20 μg) where no binding was observed when using either buffer system. Each gel image also includes a molecular weight size standard and a fixed amount of Alexa-IPD072 equivalent to a percentage of the amount needed for a 50 nM binding reaction. Gaps in gel images in panels (C) and (D) reflect noncontiguous lanes from the same gel being shown.

Incubation of WCR BBMVs (20 μg) with Alexa-IPD072 (20 nM) in the absence and presence of increasing concentrations of unlabeled IPD072Aa revealed specific binding of IPD072Aa that corresponds to relatively low binding affinity, showing a half-displacement concentration value (EC_50_) of 714 ± 120 nM unlabeled protein (Fig. 2A and B). It should be noted that IPD072Aa exists as a natural dimer in solution and the concentration for binding assumes binding of the monomer, because protein concentrations were estimated by Coomassie staining after separation by SDS-PAGE using a BSA standard curve (see Materials and Methods). Cross-linking of IPD072Aa at primary amines using bis-(sulfosuccinimidyl) suberate to prevent dissociation of the dimer revealed decrease in the apparent binding affinity (i.e., increased EC_50_ value) which is consistent with the idea that binding of the monomeric form occurs (data not shown). Although unlikely considering that Alexa-labeled IPD072 can bind with labeling on primary amines, we cannot rule-out the possibility that the cross-linking reagent could itself interfere with binding. Nevertheless, the point at which dimer dissociation might occur has not been clearly established; therefore, it remains possible that the EC_50_ value, as measured, overestimates the true EC_50_ value since concentrations were determined under denaturing conditions.

### Binding of IPD072Aa is absent from ECB BBMV.

To better understand what might contribute to the spectrum of insecticidal activity of IPD072, binding in WCR BBMVs was compared side-by-side to binding in BBMVs prepared from European corn borer (*Ostrinia nubilalis*; ECB) midguts, one of several insect species where no bioactivity was observed when tested in artificial diet bioassays (7, 10). Good specific binding was observed in WCR BBMVs as shown by the ability of saturating concentrations of unlabeled IPD072Aa to displace the binding of Alexa-IPD072 (Fig. 2C; PBS), similar to the specific binding that appears in Fig. 2A. However, no binding was observed using ECB BBMVs (Fig. 2D). Because the lepidopteran midgut is very alkaline (11), while the WCR midgut is slightly acidic (12), the binding of IPD072Aa was reevaluated under alkaline conditions with WCR and ECB BBMVs using a carbonate-based binding buffer, pH 9.6. No binding of Alexa-IPD072 was detected in either tissue, which indicates that alkaline conditions are not favorable for IPD072Aa binding (Fig. 2C and D; carbonate). Overall, these results indicate that IPD072Aa does not recognize a receptor target in ECB BBMVs and high pH is detrimental to IPD072Aa binding in WCR BBMV where a receptor target has been established under conditions that favor its binding.

### Site of action (SoA) of IPD072Aa is different than Gpp34Ab1/Tpp35Ab1 and Cry3.

**FIG 3:**
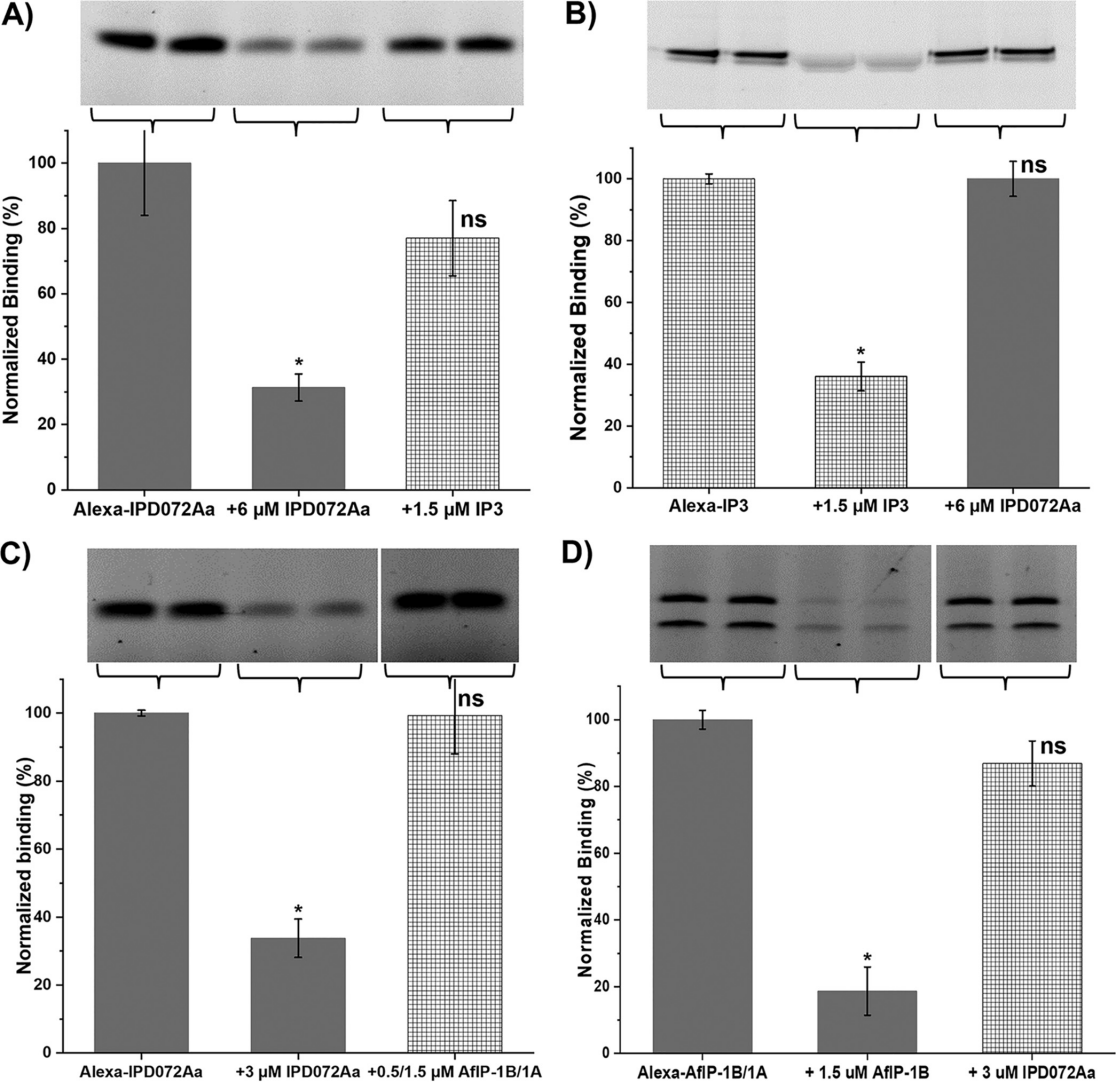
Heterologous competition between IPD072Aa and IP3-H9 (IP3) or AfIP-1A/AfIP-1B reveals no shared binding sites. Panels (A) and (C) show representative gels from competitive binding experiments using Alexa-IPD072 to evaluate the ability of saturating concentrations of unlabeled proteins to displace the labeled proteins. In each gel, the left two lanes reflect the binding of Alexa-IPD072 (20 nM) to WCR BBMVs (20 μg) with the middle two lanes reflecting homologous competition and the right two lanes reflecting heterologous competition by saturating concentrations of unlabeled IP3 (A) or AfIP1A/1B (C). Note that the signal remaining in the presence of saturating unlabeled protein during homologous competition (middle two lanes) reflects nonspecific binding of the labeled protein. Bar graphs below each gel represent the densitometry values measured from digital images of the in-gel fluorescence from multiple experiments. The values were normalized to the signal measured in the absence of unlabeled protein and are reported as the mean and SD from at least 3 replicates. Panels (B) and (D) show representative gels from competitive binding experiments using Alexa-IP3 or Alexa-AfIP-1B/AfIP-1A to evaluate the reciprocal competitions. For these competitions, Alexa-IP3 (3 nM) or Alexa-AfIP-1B (2.5 nM)/AfIP-1A (100 nM) binding to WCR BBMVs (20 μg) were tested by saturating concentrations of unlabeled IPD072Aa (6 or 3 μM, respectively). Bar graphs below each gel represent the densitometry values as described for panels (A) and (C) and are reported as the mean and SD from at least 3 replicates. Averaged densitometry values measured with homologous competitions were significantly reduced compared to the values measured in the absence of competitor (Student's *t* test; *P* < 0.001, *), while averaged densitometry values measured with heterologous competitions were not significantly different than the values measured in the absence of competitor (Student's *t* test; *P* > 0.05; ns, not significant). Gaps in gel images in panels (C) and (D) reflect noncontiguous lanes from the same gel being shown.

Several studies have reported that traits based on Cry3 proteins, whether derived from Cry3A or Cry3B are cross-resistant with each other (reviewed in reference 18), which is consistent with these proteins binding to the same receptor in WCR midguts. Additionally, WCR that are resistant to mCry3A were found to have reduced ability to bind mCry3A and a homologous WCR-insecticidal protein named IP3-H9 (10). To evaluate whether IPD072Aa shares any receptors with Cry3A proteins, heterologous competitions between IPD072Aa and IP3-H9 protein were tested. Heterologous competition of IPD072Aa binding by IP3-H9 demonstrated that these proteins do not share binding sites (Fig. 3A). In the reciprocal competition, binding of Alexa-IP3-H9 was not displaced by a saturating concentration of IPD072FL confirming that each protein binds to different receptors in BBMVs (Fig. 3B).

### Gpp34Ab1/Tpp35Ab1 SoA.

The binary protein, Gpp34Ab1/Tpp35Ab1, represents a different mode of action compared to Cry3-based traits (13, 14), which has allowed it to be utilized in pyramids for CRW control. It is important that new insecticidal proteins for trait development have a mode of action that is different from Gpp34Ab1/Tpp35Ab1, also. To evaluate this possibility, we performed competitive binding studies between IPD072, and a Gpp34Ab1/Tpp35Ab1 surrogate known as AfIP-1A/1B that binds to Gpp34Ab1/Tpp35Ab1 binding sites (6). Heterologous competition of IPD072Aa binding by AfIP-1A/1B demonstrated that these proteins do not share binding sites (Fig. 3C). In the reciprocal competition, binding of Alexa-AfIP1A/1B was not displaced by a saturating concentration of IPD072FL (Fig. 3D), confirming that IPD072Aa does not share receptors with Gpp34Ab1/Tpp35Ab1.

### IPD072Aa is localized within the insect gut.

**FIG 4:**
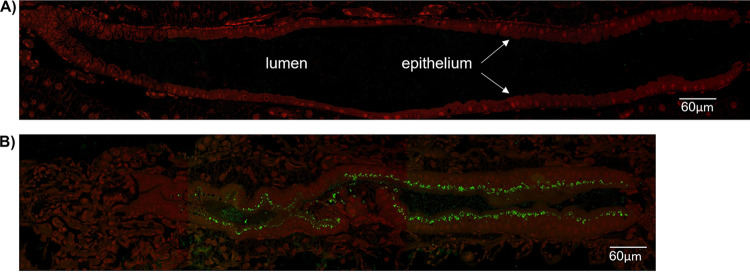
Immuno-detection of IPD072Aa in sections of WCR larvae fed on artificial diet without and with IPD072Aa. (A) The micrograph shows a longitudinal section through a WCR larva allowed to feed on control diet (without IPD072Aa) for 12 h before processing for resin embedment to allow for sectioning (see Materials and Methods). Sections were then exposed to a primary Ab directed to IPD072Aa followed by an Alexa-488 labeled secondary antibody and then evaluated by confocal microscopy. Green fluorescence was absent, but red autofluorescence of WCR tissue was observed. The dark central lumen of the gut is bordered above and below by the cells of the epithelium. (B) A representative micrograph shows a section from a larva allowed to feed for 12 h on diet that contained IPD072Aa (60 ppm) and processed identically to the control larva for microscopy. The overall size of the larva was considerably smaller than the control larva and the presence of IPD072Aa is reflected by the green fluorescence contrasted by the natural red autofluorescence of WCR tissue. In both micrographs, the anterior region of the larval section is to the right. The micrographs are reconstructions composed of multiple overlapping digital images (generated as described in Materials and Methods) to visualize the full insect sections at this magnification.

To evaluate the tissue localization and the histopathological effects of IPD072Aa on WCR larvae, insects were infested on artificial diet that contained IPD072Aa at several doses (30, 60, and 90 ppm) for various time points (12, 24, and 48 h). At each time point, larvae were collected, evaluated for feeding, and then subjected to chemical fixation to prepare for resin imbedment to allow longitudinal sections through the full length of a larva to be obtained (see Materials and Methods). Immuno-detection using fluorescence confocal microscopy revealed the presence of IPD072Aa throughout the lumen of the larval gut with intensification at enterocytes that line the gut (Fig. 4). The interaction of IPD072Aa with the enterocytes corroborates the finding of its specific binding that was observed with WCR BBMVs.

### Histopathology of IPD072.

**FIG 5:**
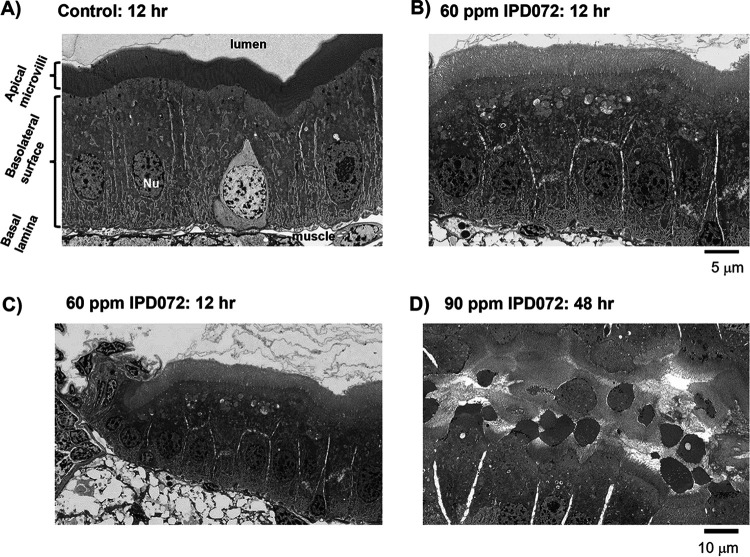
Electron micrographs showing the ultrastructure of WCR enterocytes without and after feeding on IPD072Aa. (A) The image depicts the appearance of WCR gut enterocytes of a larva that was fed on artificial diet only for 12 h. A dense layer of microvilli is apparent at the apical surface just below the lumen of gut. Other structural landmarks are labeled: the basolateral surface of the enterocytes, most of which are columnar epithelial cells; the basal lamina of the enterocytes where they incorporate into the basement membrane attaching to connective tissue and muscle surrounding the gut; and nuclei and muscle fibers are indicated. (B) Shows the appearance of the enterocytes from a larva after feeding on 60 ppm IPD072Aa for 12 h. The microvilli begin to appear slightly less dense, reflecting the loss of structural rigidity. The presence of lower density organelles become more prominent. (C) Shows a lower magnification of the same longitudinal section depicted in (B) to provide perspective over a larger section of gut, including the lumen and to give a sense of the uniformity of the effects of IPD072Aa. (D) Depicts a longitudinal section at the same magnification as the section in (C) showing the effects on a larva that fed on 90 ppm IPD072Aa for 48 h. Extensive membrane blebbing and pinching-off the apical membrane is apparent. The lumen of the gut is full of cellular debris. Dark electron dense bodies are prevalent along the plasma membrane, especially where the apical membrane appears to be preparing to separate. Dark vesicles are also present in the cellular debris within the lumen of the gut.

To evaluate the histopathology of IPD072Aa intoxication, larvae were fed on diet and processed similar to what was used for evaluating immunolocalization of IPD072Aa, but sections were processed for higher resolution electron microscopy. Larvae feeding on 60 ppm IPD072Aa for 12 h exhibit slight changes in enterocyte morphology, mostly characterized by an apparent decrease in microvilli density or rigidity and increased prominence of low-density organelles in the apical cytoplasm below the brush border membrane (Fig. 5A and B). With feeding on 90 ppm IPD072Aa for 48 h, massive membrane blebbing and pinching-off the apical membrane were observed. What appears to be remnants of enterocyte cytoplasm, some seeming to be surrounded by plasma membrane and microvilli appear throughout the lumen of gut (Fig. 5D). Dense dark vesicles are apparent at the apical plasma membrane, where blebbing is prominent, and also in the membrane debris within the gut lumen (Fig. 5D). These observations show that the pathological effects of IPD072Aa are localized within the WCR gut and lead to the conclusion that larval mortality caused by IPD072Aa can be attributed to disruption of gut function caused by enterocyte death.

## DISCUSSION

Taking into account the cross-resistance among traits based on Cry3 proteins (15), RNAi technology represents the first new mode of action for transgenic rootworm control since the approval of Cry34Ab1/35Ab1 (Gpp34Ab1/Tpp35Ab1) in 2005 (16, 17). RNAi will help to bridge a gap in resistance management prolonging the usefulness of current Bt traits (17), but there remains a sense of urgency for identifying proteins with new modes of action for trait development.

IPD072Aa was identified from P. chlororaphis, a Gram-negative bacterium that is related to Pseudomonas strains commonly used to promote plant health (18, 19). Proteins from other non-Bt bacteria that are highly active against WCR have been reported in recent years, also. Mpf3Aa1 (originally known as GNIP1Aa), a 58 kDa protein, was identified from Chromobacterium piscinae and was reported to have good activity against WCR in artificial diet bioassays (4). The structure of Mpf3Aa1 was experimentally determined and found to be a member of the MACPF family of pore forming proteins (20), but no binding or other mode of action data were reported. PIP-47Aa, a 32 kDa protein that was identified from another Pseudomonas strain, *P. mosselii*, was reported to have activity against several rootworm species (5). PIP-47Aa was shown to bind specifically to WCR BBMVs and did not share binding sites with Cry3 or Gpp34Ab1/Tpp35Ab1 proteins. AfIP-1A/1B, a binary active consisting of 16 kDa and 77 kDa proteins, was identified from the bacterium, *Alcalagenis faecalis*, and was shown to kill WCR with high potency (6). AfIP-1A/1B exhibited specific binding to WCR BBMVs at binding sites that were different from Cry3 proteins, but the same as those that bind Gpp34Ab1/Tpp35Ab1. The shared binding sites corresponded to cross-resistance to Gpp34Ab1/Tpp35Ab1 which was demonstrated using a resistant strain of WCR (6, 21). The basis of the shared binding sites and cross-resistance was illuminated when the experimental structure of AfIP-1A was solved and found to have an aegerolysin fold with strong similarity to the structure of Gpp34Ab1, another aegerolysin. These examples of non-Bt bacteria that express proteins that have high activity against WCR illustrate the potential of broader microbial screening for identifying actives for development into future traits.

The general steps in the mode of action of Bt Cry proteins have been established through a variety of studies over the course of several decades (9, 22–24). After larval ingestion, the proteins move through the alimentary canal reaching the lumen of the gut where they are solubilized, processed by proteases, bind to enterocyte membrane receptors, followed by insertion into the membrane forming pores that lead to cell death. Interaction of Cry proteins with several classes of membrane-associated proteins has been reported, including alkaline phosphatase, aminopeptidase, cadherin-like proteins, glycolipids, and ATP-binding cassette (ABC) transporters. A clear functional role has been established for cadherin-like proteins (25–27), and more recently for certain ABC transporter proteins (28, 29), even though Cry proteins that utilize these receptor targets were commercialized many years (~5 to 15 years) before functional validation occurred. Cry3 proteins have been utilized for rootworm control in transgenic maize since 2003, but a functionally validated receptor was not identified until recently when DvABCB1 was confirmed (30). The identity of the receptor for many insecticidal proteins remains to be established, including Gpp34Ab1/Tpp35Ab1. Regardless, Cry proteins target larval gut enterocytes comprising the epithelial lining causing their death which disrupts both nutritional absorption as well as the physical barrier to various pathogens ultimately leading to insect death. The results of the present study show that IPD072Aa also targets larval gut enterocytes which results in larval death. The specific binding of IPD072Aa to WCR BBMVs reveals the presence of receptors that are recognized at the gut apical membrane and the absence of binding to ECB BBMVs is consistent with the idea that the interaction is specific to the point of differential interaction with tissues from different insect orders and in alignment with its spectrum of insecticidal activity (10). Immunofluorescence microscopy revealed the localization of IPD072Aa along the lining of the larval gut associating specifically with the enterocytes (Fig. 4) that are known to be the target of Bt proteins (8). This observation corroborated the specific binding observed to WCR BBMVs and is consistent with IPD072Aa exerting its toxicity through targeting of gut enterocytes. The high-resolution EM revealed obvious changes in the ultrastructure of these enterocytes with early exposure to IPD072Aa (Fig. 5). This included an apparent decrease in rigidity and stature of microvilli and the appearance of areas of low electron density within the cytosol after a similar length of exposure where immunolocalization was assessed (12 h). With longer exposure time and higher dose, IPD072Aa caused further loss of microvilli stature and evidence of extreme disruption of the integrity of the epithelium was present. Membrane vesicles containing what appeared to be cytoplasm were observed within the lumen of the gut and signs of enterocyte detachment from the basal lamina were evident. These morphological changes reflect the toxicity of IPD072Aa to enterocytes and establishes the basis of its insecticidal activity.

IPD072Aa is a small protein composed of only 86 amino acids. Its sequence does not match any predicted structural domains, sequence signatures, or motifs when analyzed using bioinformatics software (Interpro; EMBL-EBI). Its structure was solved by NMR and found to represent a unique structural class of proteins (Poland et al., in preparation). It consists of eight beta strands organized into 2-beta sheets forming a clam shell-like array. The unique structure is consistent with IPD072Aa binding to a unique membrane receptor among the insecticidal proteins tested here. Attempts to observe pore formation by IPD072Aa in artificial lipid membranes failed to reveal pore forming activity (Nelson, unpublished observations). However, lack of pore formation *in vitro* cannot be considered deterministic as the membrane lipid composition, ionic environment, and pH were not evaluated exhaustively. Similarly, attempts to identify the plasma membrane receptor for IPD072Aa using co-precipitation and yeast two-hybrid approaches failed to identify a protein interactor that serves as a valid functional receptor (data not shown). Nevertheless, it is important to note that the specific binding to WCR BBMVs, but not to ECB BBMVs by IPD072Aa is consistent with the idea that the receptor, or at least the region where binding occurs is relatively unique. The molecular mechanism of IPD072Aa toxicity to WCR larvae, including the identity of the membrane receptor to which it binds will continue to be evaluated. However, IPD072Aa toxicity can be explained by the selective targeting and killing of rootworm enterocytes which compromises the protective and nutritional role served by the gut epithelium.

## MATERIALS AND METHODS

### Recombinant protein expression and purification.

The IPD072Aa protein with an N-terminal 6×-histidine purification tag was expressed recombinantly using an Escherichia coli expression system as described previously (7). The IP3-H9 was produced as described previously (31), as were the AfIP-1A, and AfIP-1B proteins (6). Purified proteins were dialyzed into suitable storage buffers, aliquoted, and then flash frozen in liquid nitrogen and stored at −80 C until needed.

### BBMV preparation.

The preparation of brush border membrane vesicles (BBMVs) from WCR larvae has been described previously (13). Briefly, midguts were extracted from third instars of laboratory maintained nondiapausing western corn rootworm reared on maize seedlings. Removal of the entire gut was achieved by grasping larva just behind the head capsule with forceps while grasping the anal plate region with another forceps and gently pulling. Extracted guts were immediately flash frozen in liquid nitrogen for storage at −80 C until needed. BBMVs were prepared from frozen midgut tissue similar to the method described by (32). Protein determinations were performed using the colorimetric bicinchoninic acid (BCA) method (Pierce BCA Protein assay kit, Thermo Fisher). Enrichment of apical membrane in BBMV preparations was typically 6- to 7-fold and was determined by measuring and comparing the aminopeptidase activity (enzyme associated with the apical membrane) in the final BBMV suspension to the activity in the initial crude homogenate. Aminopeptidase enzymatic activity was determined by measuring the rate of hydrolysis of the artificial substrate l-leucine-p-nitroanilide (1 mM; Sigma Cat # L2158) in 25 mM NaCl, 10 mM Tris-HCl, pH 8.0, while monitoring absorbance at 405 nm in a 96-well plate reader (Flexstation 3, Molecular Devices).

### WCR gut fluid collection.

Guts from 20 third instar WCR were extracted from the body cavity as described above and placed into a tube containing ice cold buffer (100 μL; 32 mM KCl, 1 mM CaCl_2_, 1 mM MgCl_2_, 300 mM sucrose, 5 mM Tris-HCl; pH 8.3). The tube was then vortexed briefly and gut fluid extracted by centrifugation at 20k × *g* for 15 min at 4°C. Aliquots of the gut fluid extracts were flash frozen in liquid N_2_ and stored at −80°C until needed.

### Processing of IPD072Aa by proteases.

Purified recombinantly expressed IPD072Aa protein (30 μg) was mixed with WCR gut fluid that was diluted to 20% or 30% of the extract concentration (see above) with PBS and then incubated at room temperature while under constant agitation using a microplate shaker (VWR, 12620-926) for 10 min (20%) or 120 min (30%). The percentage of gut fluid extract and time points were chosen to identify potential intermediates and final degradation products. The processing of IPD072Aa (30 μg) by purified commercially sourced proteases was evaluated in parallel by mixing with PBS-solubilized trypsin (Sigma T1426) or chymotrypsin (Sigma C3142) both at 1:50 (w:w) followed by incubation at RT while under constant agitation for 120 min. At predetermined time points, an aliquot of the IPD072Aa mixture equivalent to 15 μg of starting material was removed and mixed with protease inhibitor cocktail (cOmplete; Roche Diagnostics) and placed on ice. Once all time points were collected, the aliquots were mixed with LDS-sample buffer that contained reducing agent and heated to 100°C for 5 min and then subjected to SDS-PAGE on a 4% to 12% gel using MES buffer. The gel was then electroblotted onto PVDF membrane using an iBlot transfer system (Invitrogen, Thermo Fisher Scientific). The membrane was stained with Coomassie brilliant blue to identify bands to be cut out for Edman degradation amino acid analysis.

### BBMV binding.

Prior to binding experiments, proteins were quantified by gel densitometry following Simply Blue SafeStain (Invitrogen, Thermo Fisher Scientific) staining of SDS-PAGE resolved samples that included BSA as standards. Proteins were labeled with Alexafluor 488 (Thermo Fisher Scientific) according to manufacturer’s recommendations to track binding. To evaluate specific binding, BBMVs were incubated in binding buffer (100 μL) with Alexa-labeled IPD072Aa in the absence and presence of unlabeled protein for 1 h at room temperature with constant agitation on a high velocity orbital shaker. The binding reaction was terminated by the addition of 1 mL fresh binding buffer followed by centrifugation at 13,000 × *g* for 10 min at 4°C. The resulting pellet was washed twice by resuspending in 0.5 mL binding buffer followed by centrifugation. The final binding pellets were solubilized in 20 μL sample buffer (Novex LDS, Invitrogen), boiled for 5 min and loaded onto SDS-polyacrylamide gels (NuPage 4% to 12% Bis-Tris, Invitrogen) for resolving proteins. Nonspecific binding was defined by the amount of Alexa-labeled protein remaining in the final pellet when the binding reaction included the presence of saturating concentrations of unlabeled toxin during homologous competition binding assays. Alexa-labeled proteins were detected in-gel using a digital fluorescence imaging system (LAS4010, GE Healthcare) and quantified by densitometry software (Phoretix 1D, TotalLab Ltd., Newcastle, UK). Binding buffer consisted of 50 mM NaCl, 2.7 mM KCl, 8.1 mM Na_2_HPO_4_, 1.47 mM KH_2_PO_4_, pH 7.5 and included 0.1% Tween20 and cOmplete protease inhibitor cocktail.

Additional binding assays were performed to assess whether IPD072Aa shared binding sites with other insecticidal proteins which would indicate a high potential for cross-resistance to them. The test for competition against Cry3Aa variant, IP-3-H9 (10), was performed using the PBS binding buffer described above. The test for shared binding sites with Gpp34Ab1/Tpp35Ab1, a surrogate binary protein was used (AfIP-1A/-1B) that is completely cross-resistant and completely shares binding sites (6).

### Fixation, embedment, and sectioning of WCR neonates.

The general methods used for exposing WCR larvae to IPD072Aa and then preparing them for microscopy studies have been published previously (33, 34). Briefly, WCR eggs were applied to artificial diet as reported previously for IPD072Aa bioassay testing (7). WCR neonates were then transferred singly into wells that contained artificial diet without and with IPD072Aa at 24 h following hatch. IPD072Aa was mixed with diet at 30, 60, and 90 ppm, and free Alexafluor 488 (1.5 μM) to confirm larval feeding before processing in fixative. Larvae were collected from wells at 12, 24, and 48 h after infestation.

Neonates were collected at the specified time points and prepared for microscopy as follows. An anesthesia chamber was prepared by placing an aluminum pan filled with CO_2_ pellets in the bottom half of a vacuum desiccator and covered by the desiccator’s lid with the air valve open. Only neonates having consumed the amended diet as determined by direct observation of Alexafluor 488 fluorescence signal in the digestive tract were selected; nonfeeders were discarded. Petri plates with selected neonates were moved to the anesthesia chamber and placed on a Styrofoam block on a shelf above CO_2_ pellets for 20 min until immobile. Neonates were fixed in a solution of 5% acrolein (Sigma, St. Louis, MO) and 0.25% glutaraldehyde (Electron Microscopy Sciences, EMS, Hatfield, PA) in 100 mM cacodylate buffer pH 7.2, with 1 mM CaCl_2_ and 100 mM sucrose. Anesthetized neonates were rapidly transferred to filter paper saturated with fixation solution and additional fixation solution added until they were nearly submerged for an initial fixation of 30 min. With the aid of a dissecting microscope, an ophthalmic scalpel (EMS, Hatfield, PA) was used to remove the proximal and distal ends of each neonate to allow further penetration of the fixative and embedding resin, but without cutting through the digestive tract. Neonates were transferred to a 20-mL glass scintillation vial containing 10 mL of fresh fixation solution on a rotisserie at room temperature for 4 h, and then to a rotisserie at 4°C overnight. Neonates were rinsed 3 × 30 min in cacodylate buffer (minus the sucrose) on a rotisserie at room temperature. Samples were rinsed on a rotisserie at room temperature 3 × 30 min in cacodylate buffer, then in ddH_2_O, and transferred to 5% ethanol in ddH_2_O overnight. Dehydration continued using 20%, 50%, 80% ethanol, and three rinses in 100% ethanol, 1 h on a rotisserie at room temperature for each change. Resin embedding was achieved by infiltration with LR White (Sigma, St. Louis, MO) in ethanol at 5%, 20%, 50%, 80%, and three changes at 100%, 24 h between changes, on a rotisserie at room temperature. Infiltrated neonates were placed into gelatin capsules (Ted Pella, Redding, CA) which were then filled with fresh LR White, capped, and polymerized at 60°C for 72 h.

### Immunolocalization.

Samples for immunofluorescence localization were prepared by cutting 750 μm thick sections that were then mounted on Exell adhesion slides. Primary antibody was polyclonal antibody raised in rabbits against the recombinant full-length IPD072Aa protein expressed in E. coli, affinity purified on immobilized recombinant IPD072Aa (7). Primary antibody was prepared at final concentration of 5 μg/mL. Secondary antibody was goat anti-rabbit Alexa-488 (Molecular Probes, Eugene, OR), prepared at final concentration of 40 μg/mL. Incubation buffer for immunofluorescence localization was 20 mM Sorensen’s phosphate buffer, pH 7.2, with 150 mM NaCl and 0.2% (wt/vol) Aurion BSA-c (EMS). Sections were first incubated 30 min in 50 mM glycine to block any residual aldehyde residues, then 30 min in Aurion blocking solution for goat secondary antibody (EMS), before rinsing 6 × 5 min in incubation buffer. Primary antibody, plus no-primary control samples, were incubated 2 h at room temperature, rinsed 6 × 5 min in incubation buffer, followed by incubation in secondary antibody for 2 h at room temperature. Sections were rinsed 3 × 5 min in 20 mM Sorensen’s phosphate buffer, then 3 × 5 min in ddH_2_O, then mounted in ProLong Gold antifade (Invitrogen) and allowed to cure 24 h in the dark. Sections were imaged on a Zeiss LSM780 confocal/multiphoton microscope using a 40× objective lens (Plan-Apochromat 1.4 NA Oil) and factory preset parameters for two-channel linear unmixing of 488 nm excitation laser wavelength to separate Alexa 488 emission (~510 nm) from tissue autofluorescence (>610 nm). Several overlapping digital images were captured spanning the entire sections using Zen Blue software (Zeiss) to enable reconstruction of the full sections. Any vertical lines in the reconstructions were the result of merging the individual images. Reconstructed images were cropped and labeled using Adobe Photoshop (v23.3).
